# First molecular report of *Moniezia expansa* in small ruminants of Pakistan with epidemiological insight

**DOI:** 10.1371/journal.pone.0314343

**Published:** 2024-12-05

**Authors:** Hira Muqaddas, Naunain Mehmood, Maher Nigar, Farhana Yousaf, Kainat Farooq Khokhar, Saba Kousar, Mahnoor Aslam, Zafar Iqbal Khan, Ioannis A. Giantsis, Ayman A. Swelum, Furhan Iqbal

**Affiliations:** 1 Department of Zoology, The Women University Multan, Multan, Pakistan; 2 Department of Zoology, University of Sargodha, Sargodha, Pakistan; 3 Department of Botany, University of Sargodha, Sargodha, Pakistan; 4 Faculty of Agriculture, Department of Animal Science, Forestry and Natural Environment, Aristotle University of Thessaloniki, Thessaloniki, Greece; 5 Department of Animal Production, College of Food and Agriculture Sciences, King Saud University, Riyadh, Saudi Arabia; 6 Institute of Zoology, Bahauddin Zakariya University, Multan, Pakistan; Universidade Federal de Minas Gerais, BRAZIL

## Abstract

The members of genus *Moniezia* are the common parasites of livestock in tropical areas. The tapeworm, *Moniezia expansa* is commonly found in the gastrointestinal tract of the small and large ruminants. The present study focused on reporting the prevalence of *M*. *expansa* in small ruminants of southern Punjab: sheep and goats, in relation with epidemiological factors like age and gender. An overall prevalence of 27.2% was estimated for the small ruminants with higher infection rates in males (29.8%) and younger age group (<1 year; 32.9%). Moreover, the molecular characterization and phylogenetic analysis of the isolates based on partial *cox1* gene indicated the placement of these sequences in the *M*. *expansa* cluster. Two distinct haplotypes, without any host tropism, were identified within the Pakistani isolates. A meta-analysis for *M*. *expansa* was run for all available global reports exhibiting an overall pooled prevalence of 21.3% (CI 95%: 13.5–29.0). Additionally, a global dataset encompassing 59 partial *cox1* sequences submitted from different geographical locations was also assessed. Moderate haplotype diversity (0.760 ± 0.051) and significantly negative deviations from neutrality were estimated. The median joining haplotype network for these sequences revealed an interesting population structure indicating highly divergent sequences from China and Iraq compared to Pakistan, India, Vietnam, Senegal and Ethiopia. Given inconsistencies in genetic data there is a dire need to carry out molecular studies across the entire distributional range of *M*. *expansa* to delineate genetic diversity and population structure of the species. This will also be crucial in reevaluating the taxonomy of genus *Moniezia*.

## Introduction

*Moniezia* species have cosmopolitan distribution inhabitating the intestinal tract of ruminants [1] and are classified as members of the Anoplocephalidae family within the Cyclophyllidea order [2]. *Moniezia* spp. have typical cestode body structure; scolex, neck followed by long chained strobila. Four large suckers on the scolex act as holdfast organs for the attachment to the host [3,4]. It is distinguished by having suckers devoid of spines and by lacking rostellar hooks and rostellum [5].

*Moniezia*’s life cycle is indirect, using oribatid mites as intermediate hosts that usually thrive unhindered in grass and soil [6–8]. Domestic animals ingest the oribatid mites that are infected with the eggs of *Moniezia*. These eggs become infectious larvae (cysticercoids) which infect domestic ruminants upon ingestion [7,9]. Larvae then migrate to the ruminant’s small intestine, attach with their suckers, and develop into adult tapeworms [10,11] and are responsible for the onset of monieziasis, the gastrointestinal disorder [2].

These tapeworms are typically thought to have little pathogenicity, particularly in adult livestock, but they can cause significant illness in calves and lambs, leading to economic losses in stockbreeding [12,13]. However, a heavy infection frequently results in unfavorable clinical manifestations like pot-belly, reduced development rate, diarrhea, anemia, intestinal disease, poor quality wool, fleshless condition, and even death of the ruminant host [3,14].

Despite the significance of these tapeworms, little is known about their ecology, evolutionary biology, and population genetics. Morphological identification of these species is not easy, so far at least 12 species of the genus *Moniezia* have been identified in both domestic and wild ruminants, largely characterized by a limited set of physical traits [15]. However, these traits are convergent, leading to ongoing debates over the taxonomy of this genus [2], although genetic data are only known for three species: *Moniezia expansa*, *Moniezia benedeni*, and *Moniezia monardi* [16]. Among these, *M*. *expansa* is commonly distributed worldwide with varying regional prevalence. For instance, Alberfkani et al. [11] reported 16% prevalence from Iraq, while Bashtar et al. [4] from Egypt observed significantly higher prevalence (74%) in sheep. Most studies on the prevalence of *M*. *expansa* have relied on faecal [17,18] and post mortem examination [11,19–21]. A few studies employed multilocus enzyme electrophoresis and isoenzyme electrophoresis method to genetically compare the *Moniezia* spp. [22,23]. Similarly, few studies have employed the molecular markers (SSU rDNA, ITS1and ITS2, *cox1* and *nad1*) for the correct identification of the *M*. *expansa* and *M*. *benedeni* [2,12,24–26]. Nonetheless, due to existence of cryptic species further studies are needed to clarify the genetic diversity of *Moniezia* spp. around the world [2].

Pakistan is an agricultural country and livestock production significantly contributes to the sustenance of farmers by providing food, revenue and employment. Pakistan has a large livestock population and the prevalence of *M*. *expansa* is reported from both small and large ruminants (0.86–17.7%) [21,27,28]. However, nearly all the studies have reported prevalence via fecal examination and no published molecular report is available till date.

The aim of the present study was to investigate the epidemiological factors and molecular identification of the intestinal tapeworm, *Moniezia expansa* in small ruminants from Punjab, Pakistan, by utilizing the partial *cox1* gene. In addition to this, global overview about prevalence of this parasite and its genetic diversity analysis were performed on a global dataset for the partial *cox1* gene sequences.

## Materials and methods

### Sample collection

**Fig 1:**
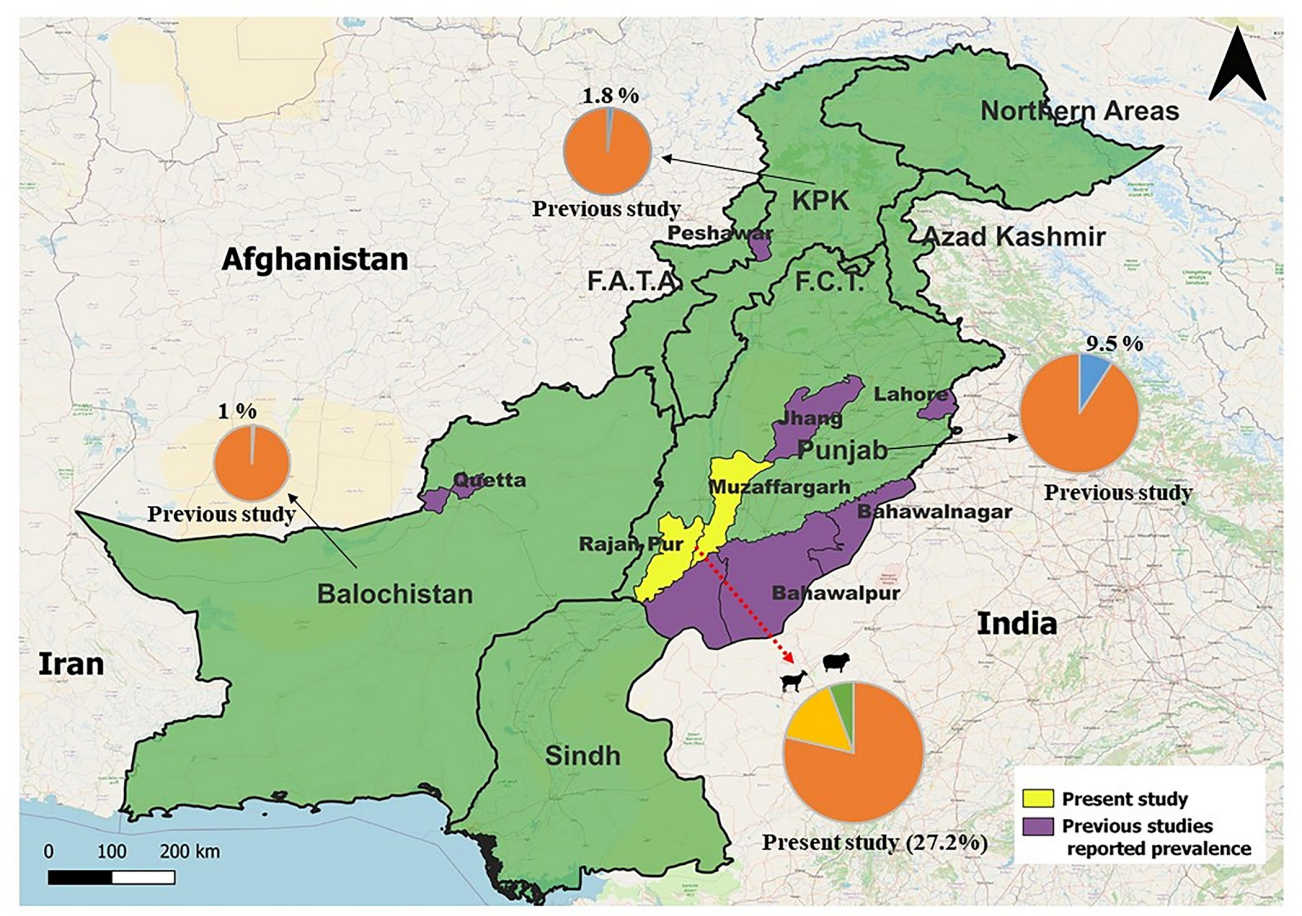
Map of Pakistan showing sampling location and prevalence from present study and estimated pooled prevalence from different provinces (based on previously published data).

Present study involved the collection of tapeworm infected small intestines (n = 464) from sheep (n = 144) and goats (n = 320) from various slaughterhouses in the Muzaffargarh and Rajanpur districts of Punjab, Pakistan (Fig 1). As the tapeworm samples were collected from slaughterhouses, hence ethival approval was not required. When available, tapeworms were removed from infected small intestines by using the forceps and washed with the saline solution. Tapeworms were stored in sterile labelled bottles with 5 mL of 70% ethanol. The tapeworms were identified on the basis of morphological characters described for *Moniezia* spp [29]. Epidemiological risk factors like age and gender of host and parasitic burden per animal (total number of tapeworms found) were noted at the time of sample collection, along with documenting the length and width of the parasite. A total of 80 worms were selected for calculating the length and width.

### Molecular analysis

The parasite tissue samples were rinsed thrice with phosphate buffered saline (PBS) to remove ethanol in which the tapeworms were preserved. Genomic DNA was extracted by using commercial kit (Gene JET Genomic DNA Purification kit, ThermoScientific, USA) according to the given instructions. PCR amplification targeted a partial mitochondrial gene encoding cytochrome c oxidase subunit 1 (*cox1*) using the primer pair JB3/JB4.5 [30] following the established protocol as reported previously by Muqaddas et al. [31]. The amplicons (450 bp) were separated on ethidium stained agarose gel (1.5%) which was later visualized and digitally captured by UV transilluminator gel documentation system (UVidoc, UK). Gel extraction kit (GeneJET, ThermoScientific, USA) was used to purify positive amplicons, which were later commercially sequenced (1st Base, Malaysia) using both forward and reverse primers as used during the PCR.

The obtained raw sequences were examined using FinchTV viewer (Geospiza, Seattle, WA, USA) to identify vague bases and check the peak quality. The obtained sequences were subsequently compared using the Basic Local Alignment Search Tool (BLAST) in the NCBI (National Centre for Biotechnology Information) database. Multiple sequence alignment of sequences obtained in the present study and those of comparable sequences downloaded from NCBI was performed in ClustalX2 software. Inter and intraspecific phylogenies have been assessed through MEGA11 software by using the Maximum Likelihood method based on the best model [32].

### Analysis of genetic diversity of *M*. *expansa*

All available nucleotide sequences for *M*. *expansa* (partial *cox1* gene) from various countries around the world were retrieved from NCBI. In order to analyze the global haplotypes analysis, a final dataset of 59 sequences was trimmed to equal base length of 317 bp, which included sequences generated from the present study and those from other regions of the world. To display the haplotype network, PopART software was used by keeping the statistical parsimony [33]. Furthermore, for global genetic diversity analysis, such as DNA polymorphism, variable sites and their number and population diversity, number of haplotypes (hn), haplotype diversity (hd) and nucleotide diversity (nd) were estimated. The neutrality indices like Tajima’s *D* and Fu’s Fs were also computed by using DnaSP 6 [34].

### Meta-analysis for *M*. *expansa*

Data from 94 articles reporting prevalence of *M*. *expansa* either through fecal examination or postpartum inspection was entered in Microsoft Excel 2016, and the detailed characteristics of each study included: region, country, host species, diagnostic method, parasitic species and reported prevalence. Later, estimated pool prevalence (%) based on 95% CI and heterogeneity (I^2^%) was estimated through OpenMetaAnalyst software.

### Statistical analysis

The descriptive statistical tool was used for estimating prevalence rates among the hosts, gender and age groups. Prevalence, estimated in percentage, was computed with the number of infected animals relative to the examined animals [35]. To establish the role of different epidemiological factors with the prevalence of monieziasis, Chi square statistic (χ^2^) was employed. All data were analyzed through SPSS (version 25) at significance level of 0.05.

## Results

### Prevalence of *Moniezia* spp. in South Punjab, Pakistan

The tapeworms belonging to genus *Moniezia* were detected in 126 out of 464 small ruminants during GI tract examination, with an overall prevalence of 27.2% (Table 1). The prevalence of *Moniezia* spp. was further determined in two age groups. The animals in the age group <1 year had the highest prevalence (32.9%), while those in the age group >1 year had the lowest prevalence (10.7%). With regards to gender, higher prevalence was recorded among males (29.8%) than females (20.9%). The mean burden of *Moniezia* spp. was three (n = 3±2.98) parasites per host with a maximum number of 12 worms retrieved from a 6 month old male goat. Moreover, the average length of worms was 167.6±2.37 cm with the mean width of 1.1± 0.27cm.

**Table 1:** Prevalence of *M*. *expansa* in different age group and gender of host species.

Variables	Category	Examined	Infected	Prevalence %	Chi-square (χ^2^)	Significance value (*p*)
Host	Goat	320	92	28.7	1.326	0.250
	Sheep	144	34	23.6		
Age group	<1	343	113	32.9	22.28	0.001 ***
	>1	121	13	10.7		
Gender	Male	325	97	29.8	3.97	0.46
	Female	139	29	20.9		
**Total**		464	126	27.2		

P > 0.05 = Non significant; P < 0.001 = Highly significant (***).

### Phylogenetic analysis of present study

**Fig 2:**
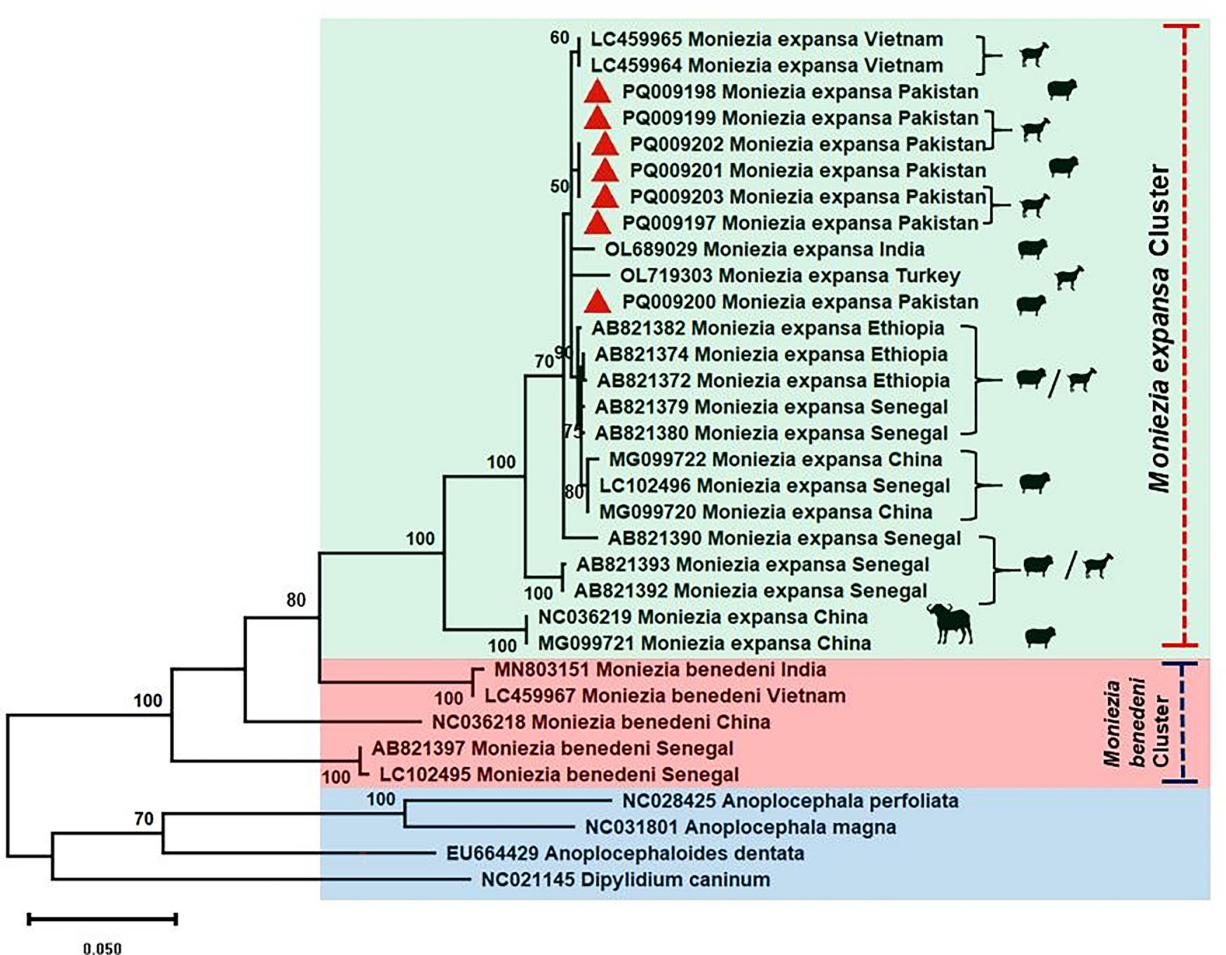
A maximum likelihood tree of *M*. *expansa* constructed from sequences of mtDNA *cox1* gene (392bp) displaying distinct *M*. *expansa* and *M*. *benedeni* clades. Bootstrap values are shown as numbers on the nodes.

Overall, 25 *Moniezia* tapeworms were sequenced for the partial *cox1* gene (392bp) which resembled with *M*. *expansa* after BLAST analysis. Two distinct haplotypes were found with no genetic distinction for sheep or goat. Among these, only seven isolates, based on host and geographical location, were submitted to GenBank (accession numbers PQ009197-203). The sheep sampled from Muzaffargarh only harbored a single dominant haplotype of *M*. *expansa* whereas, the goats harbored both haplotypes. For phylogenetic analysis, *M*. *benedeni* and other Anoplocephalid species were chosen along with *Dipylidium caninum* as an outgroup. Across phylogenetic analyses, the studied sequences fell into the clade of *M*. *expansa* (Fig 2), and showed a high similarity to *M*. *expansa* from Vietnam (LC459964-65) and India (OL689029).

### Haplotype analysis of *M*. *expansa* world population

The polymorphism analysis of global data set (n = 59; 317bp) revealed the presence of 16 haplotypes characterized by 9 singleton variable sites and 50 parsimony informative sites (Table 2). Genetic diversity estimates for *M*. *expansa* isolates revealed moderately high haplotype diversity (0.760±0.051) accompanied with low nucleotide diversity (0.0196±0.0059). A significantly negative trend was estimated for Fu’s Fs (-0.253, *p <* 0.05), whereas Tajima’s *D* exhibited negative and significant trend (-1.8996, p< 0.05).

**Table 2:** Diversity and neutrality indices for *M*. *expansa* population originating from different countries of the world.

Amplified gene	Cox1 (317bp)
No of isolates	59
No. of polymorphic sites	65
Singleton variable sites	9
Parsimony informative sites	50
Parsimony informative sites (two variants)	45
Parsimony informative sites (three variants)	4
No of Haplotypes	16
K = Average number of pairwise nucleotide difference	6.23144
Haplotype diversity (Hd) ± SD	0.760±0.051
Nucleotide diversity (π) ± SD	0.01966 ±0.00594
Tajima’s *D*	-1.89968, *p*< 0.05
Fu’s Fs	-0.253, *p* < 0.05

P < 0.05 = Least significant.

**Fig 3:**
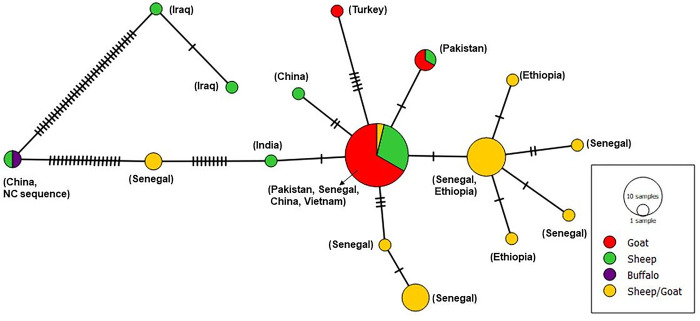
Overall haplotypic profile of *M*. *expans*a populations based on partial *cox1* (317bp) gene from different countries and their associated hosts. Hatch marks represent the number of mutations between each haplotype and the size of circle corresponds to the frequency of each haplotype in the population.

The parsimony haplotype network for *M*. *expansa* global data set showed a star shaped topology with most prevalent and centrally located haplotype from Pakistan, Senegal, China and Vietnam (Fig 3). Whereas the second most common haplotype with single mutation from centrally located haplotype is from Senegal and Ethiopia. Two haplotypes from Iraq showed large number of mutations from the central haplotype. Similarly, the reference sequence NC036219 from China is distantly related to central haplotype with large number of mutations in a small conserved region of mitochondrial gene *cox1*.

### Meta-analysis

**Fig 4:**
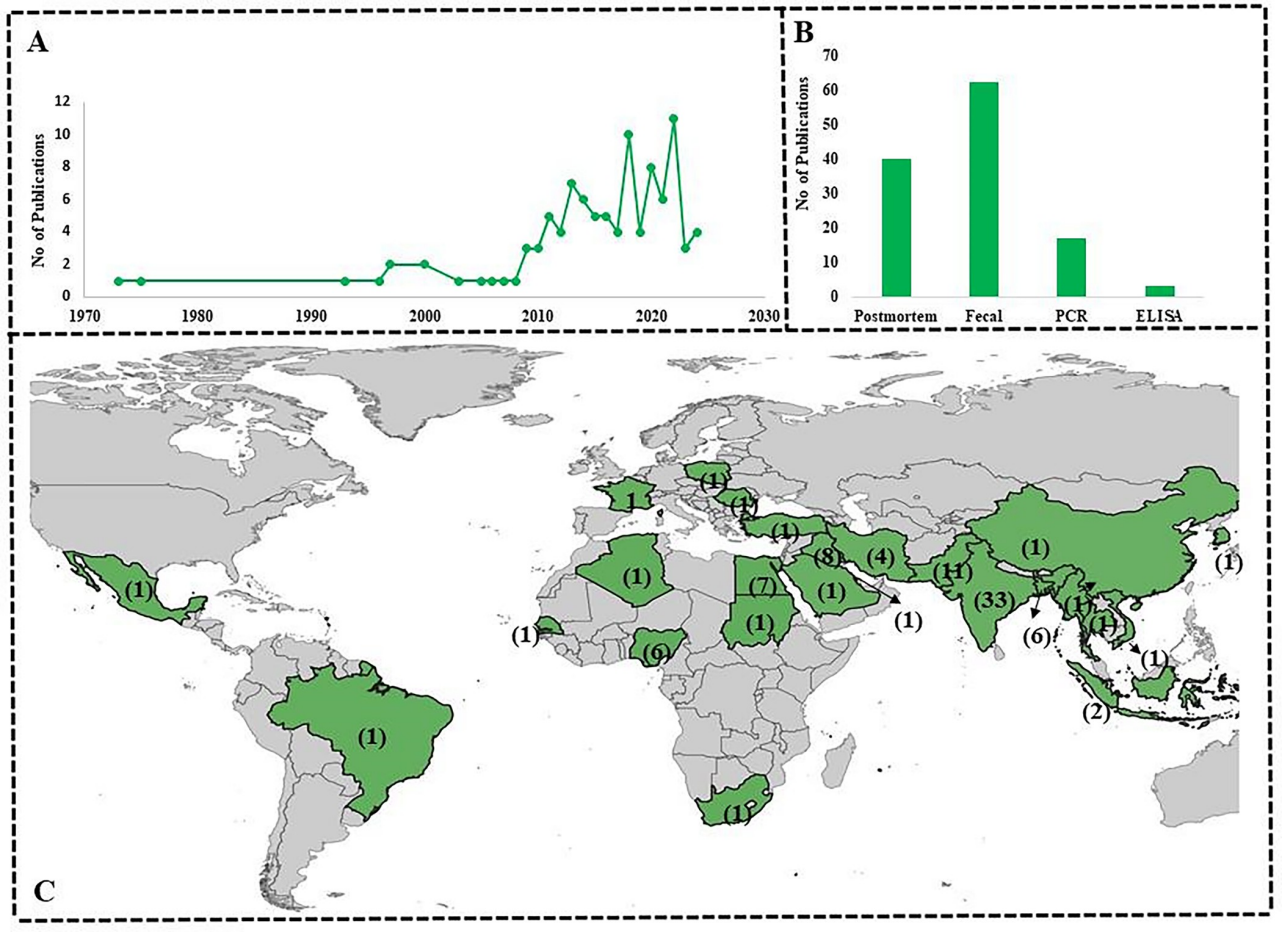
Graph illustrating the number of publications per year on *M*. *expansa* (A), techniques used for identification (B), The world map showing the number of studies conducted in various countries on the prevalence of *M*. *expansa* (C).

After review of the global literature, it seemed that monieziasis is quite common in ruminants of the tropical region. Prevalence reports mostly came from countries in Asia (n = 72), followed by few reports from Africa (n = 17), Europe (n = 3) and America (n = 2). There were great variations in prevalence between various countries and continents with an overall estimated pooled prevalence of 12.4% (CI: 95%, 10.9–13.8) reflecting diverse epidemiological dynamics within the region and countries. Among the countries in Asia, Vietnam and Iraq exhibited the highest estimated pooled prevalence of 21.5% (CI: 95%, 15.8–27.2) and 21.3% (CI: 95%,13.5–29.0) respectively. With regards to South Asia, highest number of studies (n = 33) are reported from India, in total, 23837 animals were tested and 1908 were found positive, giving rise to an estimated pooled prevalence of 9.2% (CI: 95%, 7.7–10.6%) (Table 3; Fig 4) followed by Pakistan with 11 studies with 257 positive animals with estimated pooled prevalence of 5.2% (CI: 95%, 3.0–7.4). Within Pakistan, a notable disparity in the prevalence rates was found across the provinces, with Punjab having the highest pooled prevalence (9.5%) and Baluchistan harboring the lowest pooled prevalence (1%) (Table 4).

**Table 3:** *M*. *expansa* prevalence in animals from the main continents and their countries.

Parameter	No. data sets	No. tested	No. positive	Pooled estimate % based on 95% CI	Heterogeneity I^2^%
**Overall**	94	48828	5551	12.4 (10.9–13.8)	99.09
**Africa**	**17**	**6368**	**2276**	**19.8 (7.8–31.8)**	**99.73**
Egypt	7	3962	1980	26.8 (-3.4–57.1)	99.84
Algeria	1	116	60	51.7 (42.6–60.8)	NA
Nigeria	6	1452	151	11.2 (4.7–17.7)	96.52
South Africa	1	283	6	2.1 (0.4–3.8)	NA
Senegal	1	510	74	14.5 (11.5–17.6)	NA
Sudan	1	45	5	11.1 (1.9–20.3)	NA
**Asia**	**72**	**39966**	**3032**	**9.0 (8.0–10.0)**	**97.77**
Iraq	8	1675	176	21.3 (13.5–29)	98.02
Vietnam	1	200	43	21.5 (15.8–27.2)	NA
India	33	23837	1908	9.2 (7.7–10.6)	98.44
Bangladesh	6	2084	137	11.6 (7.1–16.1)	96.08
Korea	1	546	91	16.7 (13.5–19.8)	NA
Bahrain	1	170	4	2.4 (0.1–4.6)	NA
Indonesia	2	340	31	9.1 (6.1–12.2)	0
China	1	1011	167	16.5 (14.2–18.8)	NA
Turkey	1	4003	125	3.1 (2.6–3.7)	NA
Myanmar	1	380	53	13.9 (10.5–17.4)	NA
Thailand	1	185	10	5.4 (2.1–8.7)	NA
Iran	4	277	16	4.8 (1.7–7.9)	24.74
Saudia Arabia	1	1200	14	1.2 (0.6–1.8)	NA
Pakistan	11	4058	257	5.2 (3.0–7.4)	95.65
**Europe**	**3**	**362**	**111**	**33.2 (14.7–51.6)**	**93.77**
Poland	1	158	39	24.7 (18.0–31.4)	NA
France	1	118	24	20.3 (13.1–27.6)	NA
Romania	1	86	48	55.8 (45.3–66.3)	NA
**America**	**2**	**2132**	**132**	**11.9 (-4.3–28.2)**	**98.02**
Mexico	1	1823	69	3.8 (2.9–4.7)	NA
Brazil	1	309	63	20.4 (15.9–24.9)	NA

**Table 4:** Prevalence of *M*. *expansa* among different provinces of Pakistan.

Parameter	No. data sets	No. tested	No. positive	Pooled estimate % based on 95% CI	Heterogeneity I^2^%
**Overall**	11	4058	257	5.2 (3.0–7.4)	95.65
Punjab	6	2220	224	9.5 (4.5–14.4)	97.68
Khyber Pakhtunkhwa	4	1325	28	1.8 (0.7–2.8)	39.92
Baluchistan	1	513	5	1.0 (0.1–1.8)	NA

## Discussion

In the present study, genetic diversity and other epidemiological factors contributing to *M*. *expansa* prevalence were studied. Till date, only a few studies have been carried out regarding the prevalence of monieziasis in Pakistan on domestic host species. There are gaps in knowledge about the disease and no study reporting molecular epidemiology exists in literature.

*M*. *expansa* is commonly found in sheep and goats and rarely in cattle [23,36]. The present study reported 27.2% prevalence of *M*. *expansa* in the small ruminants with goats exhibiting a higher frequency (28.7%) compared to sheep (23.6%). Nearly similar prevalence of 24% was reported from India in sheep and goats [37]. However, earlier studies in Pakistan suggested only a prevalence of 0.26–5.81% in the small ruminants from different areas of the country [38,39]. The occurrence of gastrointestinal helminths is influenced by agro-climatic factors such as pasture quality and management practices, temperature, humidity, and the grazing habits of the livestock species [40]. There is an increased parasitic burden in the host species during the rainy (monsoon) season [41] due to favorable conditions for parasite propagation and larval development [42]. In gender based analysis, a higher prevalence was observed in males (32.9%) compared to females (10.7%). Similar results were reported by Zvinorova et al. [43] where a higher number of males were infected with gastrointestinal helminths owing to differential susceptibility of males due to hormonal control and genetic predisposition. A higher chance of contact due to increased browsing time may also be attributed to high infection status [44]. In the parasitic diseases, females generally have higher infection rates due to the stress of pregnancy and parturition. However, the practice of stall-feeding females during pregnancy may reduce their exposure to contaminated grazing areas [39]. Age is one of the infection determinants for monieziasis; in current investigation animals with age bracket of less than one year were found to be more infected than age greater than one year. Increased contact with parasites and favorable conditions for ecdysis of the mite species may result in higher infection rates [45]. Moreover, it is reported that *M*. *expansa* is usually more common in animals younger than 6 to 8 months, and older animals generally exhibit reduced susceptibility. After the age of two years, the animals rarely host more than one or a small number of worms [46].

The parasite *M*. *expansa* is believed to have European origin with little variation across geographical populations on the basis of isoenzyme electrophoresis [23]. Nonetheless, taxonomic uncertainties exist for the genus *Moneizia* and both *M*. *expansa* and *M*. *benedeni* represent a species complex due to the presence of cryptic species [22,23]. A characteristic diagnostic morphological feature of pattern of interproglottidal glands in *M*. *expansa* is refuted in some studies and the utilization of genetic markers is therefore necessitated for correct species identification [2,22,23]. In current study, 25 isolates were sequenced for the partial *cox1* gene and all the samples were identified as *M*. *expansa* after phylogenetic analysis. Three clades of *M*. *expansa* were identified from sheep and goats of Senegal and Ethiopia by Diop et al. [2], however, none of the current study sequences resembled sequences in these clades despite being placed among *M*. *expansa* cluster. The current study sequences of *M*. *expansa* also differed from those identified from China and were somewhat more similar to Indian and Vietnamese isolates (Fig 2). These results indicated that the genetic variation among all geographical populations of *M*. *expansa* must be studied based on longer genetic (mitochondrial and nuclear) markers to reevaluate this species complex enabling more reliable identification.

The global data set of *M*. *expnasa* based on partial *cox1* gene (317 bp) was characterized by the presence of 16 haplotypes from a total of 59 sequences submitted from China, India, Vietnam, Pakistan, Iraq, Turkey, Senegal and Ethiopia. A moderate haplotype diversity and low nucleotide diversity were identified with negative trends for neutrality indices suggesting population expansion. The median joining haplotype network for these sequences revealed an interesting population structure placing the Pakistani isolates in the middle as a major cluster from which African isolates from Senegal and Ethiopia were branching off. The isolates from China and Iraq were highly divergent from the main cluster represented by several mutational differences within the small *cox1* fragment (Fig 3). This kind of population structure reiterates the need to address the taxonomic issues within *M*. *expansa* species complex.

## Conclusion

Current study reflects upon the prevalence and distribution of *M*. *expansa* across different continents with main locales present in Asia and Africa. Apart from outlining geographical presence, these studies fail to establish the population structure as most of the studies are not supported by molecular evidence. There is a dire need to carry out molecular studies across the entire distributional range of *M*. *expansa* to delineate genetic diversity and population structure of the species. Moreover, the taxonomical controversy about the existence of cryptic species can also be resolved by keeping in view the biological features, morphology and host tropism in addition to geographical distribution. There is also a need to identify relevant morphological characters which may be able to reliably distinguish different species of the genus *Moniezia* as most of these parasites employ similar hosts.

## References

[1] Jyoti SNK, Juyal PD. Prevalence of gastro-intestinal parasites in buffalo calves from different agro-climatic zones of Punjab. J. Parasit. Dis. 2014; 38(4): 367–370. 10.1007/s12639-013-0259-8. 25320484 PMC4185032

[2] Diop G, Yanagida T, Hailemariam Z, Menkir S, Nakao M, Sako Y, et al. Genetic characterization of *Moniezia* species in Senegal and Ethiopia. Parasitol. Int. 2015; 64(5): 256–260. 10.1016/j.parint.2015.02.008. 25752566

[3] Zhao WJ, Zhang H, Bo X, Li Y, Fu X. Generation and analysis of expressed sequence tags from a cDNA library of *Moniezia expansa*. Mol Biochem Parasitol. 2009; 164(1): 80–85. 10.1016/j.molbiopara.2008.11.009. 19118581

[4] Bashtar AR, Hassanein M, Abdel-Ghaffar F, Al-Rasheid K, Hassan S, Mehlhorn H, et al. Studies on monieziasis of sheep I. Prevalence and antihelminthic effects of some plant extracts, a light and electron microscopic study. Parasitol. Res. 2011; 108(1): 177–186. doi: 10.1007/s00436-010-2060-2 20865430

[5] Atiyah KM, Azzal GY. Biological study of *Moniezia* spp. isolated from slaughtered sheep in Basrah Provence, Southern Iraq. J. Global Sci. Res. 2022; 7(4): 2227–2233. https://www.gsjpublications.com/jgsr15920064.

[6] Akrami MA, Mostowfizadeh-Ghalamfarsa R, Ebrahimi F, Moazeni M. Molecular detection of Moniezia spp.(Cestoda) in *Pergalumna* persica (Acari: Oribatida) in Iran. Syst Appl Acarol. 2014: 23(10): 1931–1939.

[7] Schuster R, Coetzee L, Putterill JF. Oribatid mites (Acari, Oribatida) as intermediate hosts of tapeworms of the family Anoplocephalidae (Cestoda) and the transmission of *Moniezia expansa* cysticercoids in South Africa. Onderstepoort J. Vet. Res. 2000; 67(1):49–55 . 10843322

[8] Roczen-Karczmarz M, Tomczuk K. Oribatid mites as vectors of invasive diseases. Acarologia. 2016; 56(4), 613–623. https://doi.org/ff10.1051/acarologia/20164143.

[9] Abdelhamid M, Vorobiev VI, Lapteva ML, Dyab AK. Combined effect of monieziosis and hypomicroelementosis on some hematological, biochemical and hormonal parameters in merino sheep. Pak. Vet. J. 2021; 41(1): 107–111. 10.29261/pakvetj/2020.068.

[10] Guo A. *Moniezia benedeni* and *Moniezia expansa* are distinct cestode species based on complete mitochondrial genomes. Acta Trop. 2017; 166: 287–292. 10.1016/j.actatropica.2016.11.032. 27923556

[11] Alberfkani MI, Albarwary AJ, Jaafar GM, Zubair AI, Abdullah RY. Molecular characterization and phylogenetic analysis of *cox 1* and ITS 1 gene fragments of *Moniezia* species isolated from sheep. Pak. Vet. J. 2022; 42(4): 566–570. 10.29261/pakvetj/2022.073.

[12] Al-Otaibi BO, Degheidy NS, Al-Malki JS. Prevalence, incidence and molecular characterization of tape worms in Al Taif governorate, KSA and the effectiveness of *Spirulina platensis* as a biological control in vitro. Saudi J. Biol. Sci. 2021; 28(11): 6272–6278. 10.1016/j.sjbs.2021.06.086. 34759747 PMC8568705

[13] Mazyad SA, el-Nemr H I. The endoparasites of sheep and goats, and shepherd in North Sinai Governorate, Egypt. J. Egypt. Soc. Parasitol. 2002; 32(1): 119–126. . 12049248

[14] Yan H, Bo X, Liu Y, Lou Z, Ni X, Shi W, et al. Differential diagnosis of *Moniezia benedeni* and *M*. *expansa* (Anoplocephalidae) by PCR using markers in small ribosomal DNA (18S rDNA). Acta Vet. Hung. 2013; 61(4), 463–472. doi: 10.1556/AVet.2013.035 23974930

[15] Schmidt GD, 1986. Key to the genera Anoplocephalinae. pp. 421–451. In: Handbook of Tapeworm identification. CRC Press, Boca Raton.

[16] Ohtori M, Aoki M, Itagaki, T. Sequence differences in the internal transcribed spacer 1 and 5.8S ribosomal RNA among three *Moniezia* species isolated from ruminants in Japan. J. Vet. Med. Sci. 2015; 77(1): 105–107. 10.1292/jvms.14-0309 25283945 PMC4349546

[17] Azrul LM, Poungpong K, Jittapalapong S, Prasanpanich S. Descriptive prevalence of gastrointestinal parasites in goats from small farms in Bangkok and vicinity and the associated risk factors. Annual Res. Rev. Biol. 2017; 16(2): 1–7. 10.9734/ARRB/2017/34932.

[18] Pilarczyk B, Tomza-Marciniak A, Pilarczyk R, Bombik E, Seremak B, Udała J, et al. A comparison of the prevalence of the parasites of the digestive tract in goats from organic and conventional farms. Animals 2021; 11(9): 2581. doi: 10.3390/ani11092581 34573546 PMC8468771

[19] El-Seify MA, Elshahawy IS, Ibrahim O, Ahamed ZK. An abattoir-based survey on the prevalence of some gastrointestinal helminths of camels (*Camelus dromedarius*) in Aswan Province, Egypt. SVU-Int. J. Vet. Sci. 2021; 4(3), 119–130. 10.21608/svu.2021.83625.1133.

[20] Ndom M, Diop G, Quilichini Y, Yanagida T, Ba CT, Marchand B. Prevalence and scanning electron microscopic identification of anoplocephalid cestodes among small ruminants in Senegal. J. Parasitol. Res. 2016; 3937292. doi: 10.1155/2016/3937292 27597893 PMC4997042

[21] Zaman MA, Sajid M, Sikandar A, Awais MM. Point prevalence of gastrointestinal helminths and their association with sex and ge of buffaloes in lower Punjab, Pakistan. Int. J. Agric. Biol. 2014; 16(6): 1229–1231.

[22] Chilton NB, O’Callaghan MG, Beveridge I, Andrews RH. Genetic markers to distinguish Moniezia expansa from M. *benedeni* (Cestoda: Anoplocephalidae) and evidence of the existence of cryptic species in Australia. Parasitol. Res. 2007; 100(6): 1187–1192. 17206509 10.1007/s00436-006-0388-4

[23] Ba CT, Wang XQ, Renaud F, Euzet L, Marchand B, De Meeüs T. Diversity and specificity in cestodes of the genus *Moniezia*: genetic evidence. Int. J. Parasitol. 1993; 23(7): 853–857. doi: 10.1016/0020-7519(93)90049-5 8314368

[24] Alfatlawi MA, Ismail YK, Ali MJ, Karawan AC, Ibadi IN. Molecular differentiation of *Thysaniezia (Helictometra)* giardi and Moniezia species based on 18s rRNA gene in small ruminants. Iraqi J. Vet. Sci. 2021; 35(1): 105–108. 10.33899/ijvs.2020.126407.1313.

[25] Tam TT, Lan NTK, Doanh PN. Morphological differences and molecular phylogenetic relationship of two tapeworm species, *Moniezia expansa* and *Moniezia benedeni* collected from domestic ruminants in northern Vietnam. Parasitol. Int. 2020; 74: 101998. 10.1016/j.parint.2019.101998. 31634630

[26] Haukisalmi V, Laaksonen S, Oksanen A, Beckmen K, Halajian A, Yanagida T, et al. Molecular taxonomy and subgeneric classification of tapeworms of the genus Moniezia Blanchard, 1891 (Cestoda, Anoplocephalidae) in northern cervids (*Alces and Rangifer*). Parasitol. Int. 2018; 67(2): 218–224. 10.1016/j.parint.2017.12.006. 29288139

[27] Shah A, Rehman N. Coprological examination of domestic livestock for intestinal parasites in Village Bahlola, District Charsaddah (Pakistan). Pak. J. Zool. 2001; 33(4). 344–346.

[28] Ijaz M, Zaman MA, Mariam F, Farooqi SH, Aqib AI, Saleem S, et al. Prevalence, hematology and chemotherapy of gastrointestinal helminths in camels. Pak. Vet. J. 2018; 38(1): 81–85. doi: 10.29261/pakvetj/2018.016

[29] Spasski AA. Essentials of cestology. Anoplocephalate tapeworms of domestic and wild animals. Vol. 1. 1961.

[30] Bowles J, Blair D, McManus DP. Genetic variants within the genus *Echinococcus* identified by mitochondrial DNA sequencing. Mol. Biochem. Parasitol. 1992; 54(2): 165–173. doi: 10.1016/0166-6851(92)90109-W 1435857

[31] Muqaddas H, Mehmood N, Arshad M. Genetic variability and diversity of *Echinococcus granulosus* sensu lato in human isolates of Pakistan based on cox1 mt-DNA sequences (366bp). Acta Trop. 2020; 207: 105470. 10.1016/j.actatropica.2020.105470. 32302687

[32] Kumar S, Stecher G, Li M, Knyaz C, Tamura K. MEGA X: molecular evolutionary genetics analysis across computing platforms. Mol. Boil. Evol. 2018; 35(6): 1547–1549. doi: 10.1093/molbev/msy096 29722887 PMC5967553

[33] Bandelt HJ, Forster P, Röhl A. Median-joining networks for inferring intraspecific phylogenies. Mol. Boil. Evol. 199916(1)37–48. doi: 10.1093/oxfordjournals.molbev.a026036 10331250

[34] Rozas J, Ferrer-Mata A, Sánchez-DelBarrio JC, Guirao-Rico S, Librado P, Ramos-Onsins SE, Sánchez-Gracia A. DnaSP 6: DNA sequence polymorphism analysis of large data sets. Mol. Boil. Evol. 2017; 34(12): 3299–302. doi: 10.1093/molbev/msx248 29029172

[35] Mehmood N, Arshad M, Ahmed H, Simsek S, Muqaddas H. Comprehensive account on prevalence and characteristics of hydatid cysts in livestock from Pakistan.Korean J. Parasitol. 2020 Apr;58(2):121. doi: 10.3347/kjp.2020.58.2.121 32418380 PMC7231835

[36] Nguyen TD, Le QD, Huynh VV, Nguyen ST, Nguyen TV, Vu-Khac H. The development of PCR methodology for the identification of species of the tapeworm *Moniezia* from cattle, goats and sheep in central Vietnam. J. Helminthol. 2012; 86(4): 426–9. doi: 10.1017/S0022149X11000629 22071022

[37] Kumar S, Kaur H. Molecular characterization of Moniezia denticulata (Rudolphi, 1810) and its distinction from *M*. *expansa* infecting sheep and goats raised in the north and north-western regions of India. Parasitology. 2023; 150(9): 831–41. 10.1017/S003118202300063X. 37555338 PMC10478047

[38] Ayaz MM, Raza MA, Murtaza S, Akhtar S. Epidemiological survey of helminths of goats in southern Punjab, Pakistan. Trop. Biomed. 2013; 30(1): 62–71. . 23665709

[39] Raza MA, Arshad HM, Ayaz MM, Bachaya SM, Basit A. Gastrointestinal helminthiasis in goats at the localities of Jatoi, Punjab, Pakistan. Sci. Int. (Lahore). 2012; 24(2): 171–5.

[40] Gupta A, Singh NK, Singh H, Rath SS. Assessment of risk factors associated with prevalence of gastrointestinal helminths in buffaloes from Punjab State, India. Buffalo Bullet. 2018; 37(3): 279–90.

[41] Pathak AK, Pal S. Seasonal prevalence of gastrointestinal parasites in goats from Durg district of Chhattisgarh. Vet. World. 2008; 1(5):136–138.

[42] Faizal AC, Rajapakse RP. Prevalence of coccidia and gastrointestinal nematode infections in cross bred goats in the dry areas of Sri Lanka. Small Rumin. Res. 2001; 40(3): 233–238. doi: 10.1016/s0921-4488(01)00179-1 11323207

[43] Zvinorova PI, Halimani TE, Muchadeyi FC, Matika O, Riggio V, Dzama K. Prevalence and risk factors of gastrointestinal parasitic infections in goats in low-input low-output farming systems in Zimbabwe. Small Rumin. Res. 2016; 143: 75–83. doi: 10.1016/j.smallrumres.2016.09.005 27766016 PMC5063533

[44] Ouattara L, Dorchies P. Gastro-intestinal helminths of sheep and goats in subhumid and sahelian areas of Burkina Faso. Revue de Médecine Vétérinaire, 2001; 152(2): 165–170.

[45] Purja R, Maharjan M. Gastrointestinal parasites in goat (*Capra hircus*) of Puranchaur VDC, Pokhara. Int. J. Res. Studies Zool. 2017; 3(4): 39–45. 10.20431/2454-941X.0304005.

[46] Mullen GR, OConnor BM. *Mites (Acari)*. In: Mullen GR, Durden LA, editors. Medical and veterinary entomology. 3^rd^ ed. New York: Academic Press; 2019. pp. 533–602.

