# Expression of Concern: First molecular report of *Moniezia expansa* in small ruminants of Pakistan with epidemiological insight

**DOI:** 10.1371/journal.pone.0354434

**Published:** 2026-07-23

**Authors:** The PLOS One Editors

The *PLOS One* Editors issue this Expression of Concern to alert readers that this article [1] was identified as one of a series of submissions for which we have concerns about adherence to the PLOS Authorship policy. We regret that these issues were not addressed prior to the article’s publication.

## References

[1] Muqaddas H, Mehmood N, Nigar M, Yousaf F, Khokhar KF, Kousar S, et al. First molecular report of *Moniezia expansa* in small ruminants of Pakistan with epidemiological insight. PLoS One. 2024;19(12):e0314343. doi: 10.1371/journal.pone.0314343 39636935 PMC11620685

